# Low plasma angiotensin-converting enzyme 2 level in diabetics increases the risk of severe COVID-19 infection

**DOI:** 10.18632/aging.202967

**Published:** 2021-05-06

**Authors:** Yanliang Zhang, Yangyang Sun, Kang Liu, Raphael N Alolga, Xueqiang Xu, Ganzhu Feng, Pingxi Xiao

**Affiliations:** 1 Department of Infectious Diseases, Nanjing Hospital of Chinese Medicine Affiliated to Nanjing University of Chinese Medicine, Nanjing, China; 2 State Key Laboratory of Natural Medicines, Clinical Metabolomics Center, Department of Pharmacognosy, School of Traditional Chinese Pharmacy, China Pharmaceutical University, Nanjing, China; 3 Department of Nephrology, First Affiliated Hospital of Nanjing Medical University, Nanjing, China; 4 Department of Respiratory Medicine, The Second Affiliated Hospital of Nanjing Medical University, Nanjing, China; 5 Department of Cardiology, The Affiliated Sir Run Run Hospital of Nanjing Medical University, Nanjing, China

**Keywords:** COVID-19, angiotensin-converting enzyme 2, diabetes

## Abstract

Patients with pre-existing chronic diseases are more susceptible to coronavirus disease 2019 (COVID-19), yet the underlying causes of increased risk are of infection remain unclear. Angiotensin-converting- enzyme 2 (ACE2), the cell surface receptor that recognizes the coronavirus spike protein has protective effects against inflammation and chronic hyperglycemia in animal models. The roles of ACE2 in severe SARS-CoV-2 infections remains ambiguous due to contradictory findings. In this study, we aimed to investigate the relationship between human plasma ACE2 levels in diabetics and the high risk of severe SARS-CoV-2 infection. First, the medical records of 245 patients with SARS-CoV-2-positive who have chronic diseases were analyzed. We also recruited 404 elderly subjects with comorbid chronic diseases such as diabetes mellitus, coronary heart disease, cerebrovascular disease, hypertension and obesity, and investigated the ACE2 plasma levels. Plasma concentrations of ACE2 were much lower (2973.83±2196.79 pg/mL) in diabetics with chronic disease than in healthy controls (4308.21±2352.42 pg/ml), and the use of hypoglycemia drugs was associated with lower circulating concentrations of ACE2 (P=1.49E-08). Diabetics with lower plasma levels of ACE2 may be susceptible to severe COVID-19. Our findings suggest that the poor prognosis in patients with diabetes infected with SARS-CoV-2 may be due to low circulating ACE2 levels.

## INTRODUCTION

The current COVID-19 pandemic remains a global menace. According to the data on 19 March 2021, it has infected more than 1.23 billion people globally with over 2.7 million deaths [1]. Early epidemiological observations have indicated that SARS-CoV-2 infection leads to deleterious hyper-inflammatory reactions [2]. Recent data suggest that patients with comorbid conditions such as cardiac diseases, hypertension, and diabetes mellitus (DM), are at increased risk of contracting severe SARS-CoV-2 infection [3, 4]. However, the underlying causes of high morbidity and mortality of these patients are unrevealed.

The coronavirus receptor angiotensin-converting enzyme 2 (ACE2), widely expressed in the lungs, liver, kidneys, and blood vessels has been recognized as a participant in SARS-CoV-2 host cell entry [5]. The distribution and abundance of ACE2 in organs may be closely correlated with population susceptibility and progression of COVID-19 [6]. ACE2 deficiency disrupts glucose homeostasis and worsens inflammation in diabetic animal models [7, 8], however whether or not the changes of ACE2 levels contribute to the susceptibility and high mortality of COVID-19 patients with chronic diseases to remains obscure.

We analyzed medical records of 245 patients with SARS-CoV-2 infection from Wuhan Jinyintan Hospital and Huangshi Hospital of Traditional Chinese Medicine in Hubei province of China, and confirmed the upregulated inflammation in these patients. With the knowledge that patients with chronic diseases are more prone to the devastating effects of COVID-19, we recruited 404 elderly subjects with comorbid chronic diseases such as DM, coronary heart disease, cerebrovascular disease, hypertension and obesity and investigated the plasma levels of ACE2. Our data indicated that lower plasma concentrations of ACE2 in diabetics may be correlated with susceptibility to severe COVID-19.

## RESULTS

### Clinical characteristics of COVID-19 patients

Characteristics of patients infected by SARS-CoV-2 are presented in Table 1. The median age was significantly positively correlated with the severity of disease. Older men were more severely infected with SARS-CoV-2. Patients with DM, hypertension and cardiovascular diseases suffered the worst outcome of the disease. Table 1 also shows the change of white blood cells (WBC), NEU and LYM with the progression of COVID-19. The median level of IL-6 was 3.17 (ng/L) in common patients (IQR, 1.50–6.38), 6.82 (ng/L) in severe patients (IQR, 2.51–15.48), and 12.90 (ng/L) in critically ill patients (IQR, 9.11–18.54). The median CRP was 24.44 (mg/L) in common patients (IQR, 6.70–58.23), 67.28 in severe patients (IQR, 41.90–77.80), and 160 in critically ill patients (IQR, 85.25–160). ALT and AST are both increased with the severity of SARS-CoV-2 infection. Our data confirm that inflammation response with massive cytokine release occurred after SARS-CoV-2 infection.

**Table 1:** Characteristics of COVID-19 patients.

**Characteristics of COVID-19 patients**				
	**Common(n=92)**	**Severe(n=40)**	**Critically ill(n=113)**	***P***
Age (median (IQR))	56.50 [46.00, 65.00]	66.50 [55.00, 81.00]	65.00 [59.00, 73.00]	**<0.001^a^**
Sex = Male (%)	44 (47.8)	24 (60.0)	77 (68.1)	**0.013^a^**
Comorbidity				
DM (%)	5 (5.4)	2 (5.0)	25 (22.1)	**0.001^b^**
HTP (%)	8 (8.7)	9 (22.5)	47 (41.6)	**<0.001^b^**
CVD (%)	3 (3.3)	5 (12.5)	8 (7.1)	0.135**^b^**
Laboratory data (median [IQR])				
WBC (×10^9^/L)	4.54 [3.50, 5.58]	5.63 [4.04, 7.36]	13.79 [10.16, 19.56]	**<0.001^a^**
IL-6 (ng/L)	3.17 [1.50, 6.38]	6.82 [2.51, 15.48]	12.90 [8.44, 17.84]	**<0.001^a^**
NEU count (×10^9^/L)	2.90 [2.18, 4.00]	4.49 [2.80, 5.93]	12.82 [9.11, 18.54]	**<0.001^a^**
LYM count (×10^9^/L)	1.00 [0.70, 1.35]	0.66 [0.54, 0.87]	0.54 [0.33, 0.84]	**<0.001^a^**
Hb(g/L)	131.00 [117.00, 140.00]	125.00 [114.25, 133.75]	114.00 [98.00, 126.00]	**<0.001^a^**
PLT (×10^9^/L)	159.50 [124.50, 201.75]	177.50 [140.75, 241.25]	148.00 [80.00, 229.00]	0.05**^a^**
ALT(U/L)	22.00 [15.00, 34.00]	29.50 [19.00, 52.00]	41.00 [20.00, 79.00]	**0.001^a^**
AST(U/L)	29.00 [22.00, 39.00]	42.00 [29.00, 50.75]	51.00 [32.00, 72.00]	**<0.001^a^**
ALB(g/L)	39.00 [35.50, 42.25]	35.05 [32.55, 38.25]	27.70 [25.70, 30.50]	**<0.001^a^**
CRP(mg/L)	24.44 [6.70, 58.23]	67.28 [41.90, 77.80]	160.00 [85.25, 160.00]	**<0.001^a^**
PCT (μg/L)	0.10 [0.06, 0.13]	0.15 [0.08, 0.29]	0.62 [0.19, 2.49]	**<0.001^a^**
BNP (pg/ml)	29.05 [11.25, 106.78]	108.20 [49.83, 433.90]	200.60 [86.25, 853.20]	**0.002^a^**
Cr(umol/L)	65.91 [50.20, 78.35]	55.64 [46.67, 70.86]	74.70 [58.45, 91.40]	**0.001^a^**

DM, Diabetes mellitus; HTP, Hypertension; CVD, Cardiovascular diseases; WBC, White blood cell; NEU, Neutrophil; LYM, Lymphocyte; Hb, Hemoglobin; PLT, Platelet count; ALT, Alanine aminotransferase; AST, Aspartate aminotransferase; ALB, Albumin; CRP, C reactive protein; PCT, Procalcitonin; BNP, B-natriuretic peptide; Cr, Creatinine; **P<0.01, ***P<0.001. ^a^Chi-square test;^b^Kruskal-Wallis test.

### Plasma ACE2 concentrations decreased in patients with diabetics

**Figure 1:**
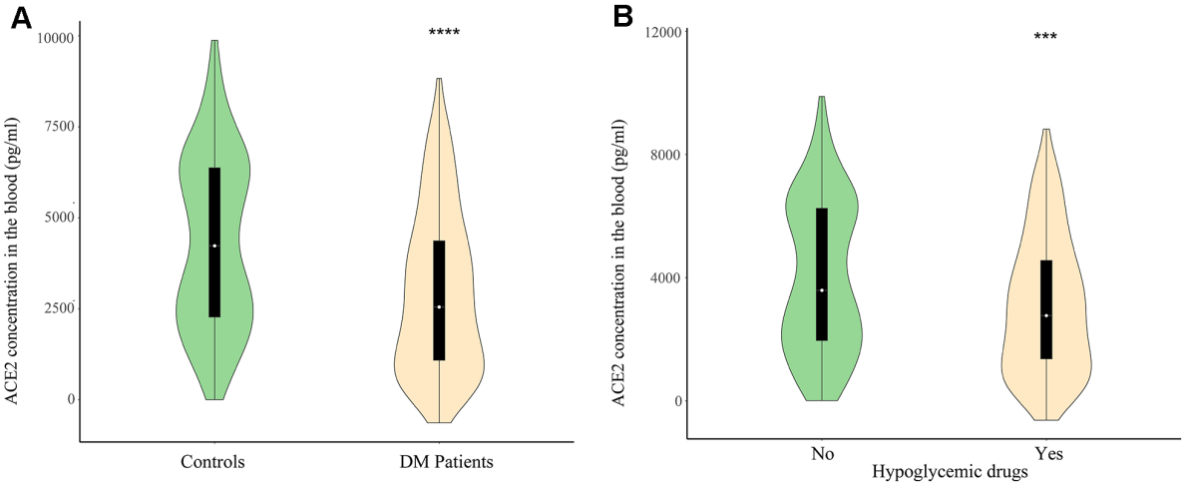
(**A**) The plasma level of ACE2 in diabetes mellitus patients and control. (**B**) The plasma level of ACE2 in users of hypoglycemic drugs and non-users. **P<0.01, ***P<0.001.

ACE2 deficiency disturbed glucose metabolism and exacerbated inflammation in diabetic animal model [7, 8], where explored the associations between plasma ACE2 and the risk for mortality in diabetics with severe SARS-CoV-2 infection. According to the HbA1c levels (HbA1c≥6.5%) and fasting blood glucose level (FBG≥7 mmol/L), all subjects were divided into DM and non-DM groups (Table 2). The median age of DM patients was 56.9 years, and 57.8 years for non-DM group (Table 2). Patients with diabetics have at least one co-morbidity (hypertension, coronary heart disease, cerebrovascular disease, and obesity). All subjects take at least one of the following medications: anti-hypertension drugs, lipid-lowering drugs, hypoglycemic drugs, or ACEIs. We found that plasma concentrations of ACE2 were significantly lower in diabetics patients (2973.83 ± 2196.79 pg/mL) than in the non-DM group (4308.21 ± 2352.42 pg/mL) (Figure 1A), which indicated that downregulated ACE2 levels were correlated with impaired glucose homeostasis in patients with diabetes. In order to evaluate whether diabetic comorbidities could affect statistical analyse, we performed a multivariate analysis of variance on these confounding factors, and the results showed that complications in diabetics have no significant impacts on the conclusion (Table 3).

**Table 2:** Characteristics of diabetes mellitus patients and controls.

**Characteristics of diabetes mellitus (DM) patients and controls**			
	**DM Patients** **(n= 174)**	**Controls** **(n= 230)**	***P***
Gender (%)			0.932
Male	99(56.9)	133(57.8)	
Female	75(43.1)	97(42.2)	
Age (mean (SD))	63.03 (11.81)	59.54 (12.43)	**0.005**
BMI (%)			0.597
≤18.5	2(1.1)	2(0.8)	
18.5-24	59(33.9)	88(38.3)	
24-28	72(41.4)	98(42.6)	
≥28.0	37(21.3)	40(17.4)	
Comorbidity (%)			
Coronary heart disease	143(82.2)	179(77.8)	0.340
Hypertension	134(77.0)	122(53.0)	**<0.001**
Cerebrovascular disease	19(10.9)	35(15.2)	0.448
Laboratory data (mean (SD))			
Glucose (mmol/L)	8.46 (3.66)	5.36 (1.03)	**<0.001**
HbA1c (%)	7.51 (1.77)	5.41 (0.63)	**<0.001**
TG (mmol/L)	2.10 (2.02)	1.49 (0.70)	**0.001**
TC (mmol/L)	4.04 (1.13)	4.21 (1.03)	0.198
HDL-C (mmol/L)	1.08 (0.35)	1.25 (0.39)	**<0.001**
LDL-C (mmol/L)	2.38 (0.99)	2.66 (0.91)	**0.012**
BUN (mmol/L)	6.33 (2.42)	5.45 (1.73)	**<0.001**
Creatinine (umol/L)	72.15 (24.66)	70.17 (18.60)	0.420
Medication history (%)			
Lipid-lowering drugs	160 (92.0)	198 (86.1)	**<0.001**
Hypoglycemic drugs	141 (81.0)	0 (0)	**<0.001**
Hypotensive drugs	158 (90.8)	193 (83.9)	**0.001**
ACEI/ABR	102 (58.6)	91 (39.6)	**<0.001**

BMI, Body mass index; HbA1c, Hemoglobin A1C;TG, Triglyceride; TC, Triglyceride cholesterol; HDL-C, High density lipoprotein cholesterol; LDL-C, Low density lipoprotein cholesterol; BUN, Blood urea nitrogen; ACEI/ABR, Angiotensin-converting enzyme inhibitor/ Angiotensin receptor blocker.**P<0.01, ***P<0.001. ^a^Chi-square test; ^b^Student’s *t* test.

**Table 3:** Effect of comorbidities in patients with diabetes on the plasma level of ACE2.

**Covariates**	**F value**	***P***
Diabetes	33.578	1.39e-08
Hypertension	0.209	0.647
Coronary heart disease	1.274	0.260

### ACE2 concentrations reduced in diabetics with hypoglycemia drugs

To investigate the causes of lower plasma concentrations of ACE2 in diabetics with chronic disease, we evaluated the influence of medications on ACE2. Figure 1B shows that plasma ACE2 is reduced in diabetics treated with hypoglycemia drugs (3103.77 ± 2211.86 pg/ml) compared to diabetics who did not receive the medication (4038.58±2439.10 pg/ml). Based on currently available evidence, we therefore hypothesize that lower plasma ACE2 increases the risk of developing severe and fatal SARS-CoV-2 infection.

## DISCUSSION

Diabetics have increasing susceptibility and risk of mortality upon SARS-CoV-2 infection. Given that patients with diabetes belong to this high-risk group, we sought to uncover potential biological factors that could explain the susceptibility to SARS-CoV-2 in this vulnerable population. Our investigation revealed that plasma levels of ACE2 were significantly lower in DM patients compared to the non-DM group. After adjusting for confounding factors including hypertension and coronary heart disease in diabetics by multivariate ANOVA analysis, we observed that hypoglycemic drug intake significantly reduced the plasma ACE2 concentration in diabetics. These results suggested that the lower plasma ACE2 levels in diabetics might increase their risk of developing severe and fatal symptoms of COVID-19.

About 20–50% of COVID-19-infected patients were diabetic, and the mortality of patients with diabetes is 50% higher than those without diabetes [9]. It had been reported that accumulation of angiotensin I (Ang-I or A [1–10]) and reduced A [1–9] concentration in patients succumbing to ARDS full name is related to mortality, which suggest that ACE2 activities may be reduced in the non-surviving ARDS patients [10]. Consistent with this perspective, our study indicated that lower plasma ACE2 in diabetics treated with hypoglycemic drugs may be correlated with higher mortality from COVID-19. However, there are controversial results regarding the value of plasma ACE2 levels in the risk of COVID-19. Recent studies show that higher plasma level of soluble ACE2 (sACE2), which are associated with a higher risk for mortality in patients with atrial fibrillation, heart failure and diabetes mellitus, might contribute to improved methods of identifying risk for severe COVID-19 infection [11–13]. Several studies have found that the use of ACEI/ARB led to an increase in circulating ACE2 levels, thus, accounting for a lower risk of all-cause mortality of COVID-19 compared to non-users [14, 15]. The mean age of all participants with heart disease included in these previous studies were elder than those in our clinical trials, and about 80% of these patients have a history of taking ACE inhibitors or ARBs, while only about 50% of patients have such history of drug intake in our investigation. As our study focuses on the plasma ACE2 level in Chinese diabetics, the possible effect of genetic variation on plasma ACE2 levels remains unclear. Differences in the demographic makeup of the patients included in the study may account for the inconsistencies between our data and those of other studies. Our findings may provide some biological context for the devastating effects of COVID-19 towards diabetics.

Some reports have suggested that ACE2 deficiency results in increased glucose intolerance, epicardial adipose tissue (EAT) inflammation and heart failure in high-fat diet induced ACE2-null mice [8]. ACE2 overexpression may ameliorate hyperglycemia in diabetic animal models [16]. Previous studies show that low plasma ACE2 levels are correlated with the severe of lung pathologies among SARS patients [17]. These findings identified ACE2 as a key negative regulator of lung edema and acute lung failure. Here, we hypothesized that reduced plasma ACE2 concentrations caused by use of hypoglycemic drugs may contribute the worse outcome in COVID-19 patients with diabetes.

The limitations of this study are as follows: Firstly, the conclusions drawn in this analysis are mainly restricted to diabetics, albeit a group of patients at high risk and mortality for COVID-19. Secondly, as included patients are not SARS-CoV-2 infected, we cannot provide a direct link between the progression of COVID-19 disease in diabetics with low plasma ACE2 concentrations, and the influence of age and hypoglycemic drug intake on the course of the disease. Thirdly, we measured plasma ACE2 concentrations, which may not be reflective of tissue levels. We can only speculate that circulating concentrations are consistent with tissue concentration, since there is no compelling evidence for this.

In summary, this study found a significantly lower plasma levels of ACE2 in diabetics compared to non-diabetics. Considering that DM patients are at a higher risk of mortality from COVID-19, and diabetes mellitus is characterized by EAT inflammation in ACE-deficient conditions [7], we suspect that the relatively low plasma ACE2 level might promote a surge inflammation and subsequent organ dysfunction following SARS-CoV-2 infection. Our findings uncover a potential explanation for the poor prognosis of DM patients infected with SARS-CoV-2.

## MATERIALS AND METHODS

### Study participants

Our study focused on PCOVID-19-infected patients with one or more pre-existing chronic conditions (cardiac diseases, hypertension, or diabetes), from Wuhan Jinyintan Hospital and Huangshi Hospital of Traditional Chinese Medicine in Hubei province of China (n= 245). Patients with and without diabetes (n= 404) have at least one co-morbidity (hypertension, coronary heart disease, cerebrovascular disease and obesity), and all subjects take at least one of the following: anti-hypertension drugs, lipid-lowering drugs, hypoglycemia drugs and ACE2 inhibitors.

### Laboratory testing

All medical laboratory data in COVID-19-infected patients, were generated by the clinical laboratory of Wuhan Jinyintan Hospital and Huangshi Hospital of Traditional Chinese Medicine. This includes numbers of white blood cells (WBC) and neutrophils (NEU); and serum concentrations of hemoglobin (Hb), platelet count (PLT), alanine aminotransferase (ALT), aspartate aminotransferase (AST), albumin (ALB), C-reactive protein (CRP), procalcitonin (PCT), B-natriuretic peptide (BNP) and creatinine (Cr). In this study, SARS-CoV-2 virus at the early stage of the outbreak of COVID-19 is very toxic, there is the high risk of manipulating these samples for ACE2 measurement. Plasma ACE2 was only measured in the cohort without SARS-CoV-2 infection.

All medical laboratory data in patients with or without diabetes, were generated by the clinical laboratory of the Affiliated Sir Run Run Hospital of Nanjing Medical University. This includes serum concentrations of hemoglobin A1C(HbA1c), triglyceride (TG), triglyceride cholesterol (TC), high density lipoprotein cholesterol (HDL-C), low density lipoprotein cholesterol (LDL-C) and blood urea nitrogen (BUN). The plasma concentration of ACE2 was detected by ELISA kit (CLOUD-CLONE CORP.).

### Statistical analysis

Continuous variables were presented using mean ± SD (standard deviation) or median and IQR (25th and 75th percentiles; interquartile range), and as the number (percent) for categorical variables. A measure of statistical significance between different variables was determined by Mann-Whitney U test or student’s t test. The χ2 test for categorical variables was used to examine the difference between groups. Multivariate ANOVA analysis was performed for further adjusting confounding factors. All tests were two-sided, and P < 0.05 was considered statistically significant unless stated otherwise.
